# Synchronous Bilateral Carotid Body and Bilateral Vagal Paragangliomas: A Case Report and Literature Review

**DOI:** 10.3390/jcm15124463

**Published:** 2026-06-09

**Authors:** Nahar Alanezi, Yazeed Alofisan, Mohammed Dahman, Hassan Gado, Majid Althobaiti, Omar Tawfiq Abualnasr, Tariq Alanezi

**Affiliations:** 1 Department of Surgery, College of Medicine, Imam Mohammad Ibn Saud Islamic University (IMSIU), Riyadh 11564, Saudi Arabia; 2 Department of Surgery, Division of Vascular Surgery, Security Forces Hospital, Riyadh 11564, Saudi Arabia; yalofisan@moi.med.sa (Y.A.); hasanvascular@yahoo.com (H.G.); 3 Division of Vascular Surgery and Endovascular Therapy, Department of Surgery, King Faisal Specialist Hospital and Research Center (KFSH&RC), Riyadh 12713, Saudi Arabia; m.mdahman@hotmail.com; 4 Department of Otolaryngology-Head and Neck Surgery, Security Forces Hospital Program, Riyadh 11564, Saudi Arabia; consult@gmail.com; 5 College of Medicine, Alfaisal University, Riyadh 11533, Saudi Arabia; midotawfiq@gmail.com; 6 Division of Vascular Surgery, Department of Surgery, University of Toronto, Toronto, ON M5S 1A1, Canada; tariq.alanezi@mail.utoronto.ca

**Keywords:** head and neck paraganglioma, carotid body tumor, vagal paraganglioma, multicentric disease, catecholamines, complex vascular tumor, genetic testing

## Abstract

**Background:** Head and neck paragangliomas (HNPGLs) are rare neuroendocrine neoplasms that may be sporadic or hereditary and may occur as isolated, multifocal, or syndromic disease within the pheochromocytoma-paraganglioma spectrum. Synchronous bilateral carotid body tumors (CBTs) and bilateral vagal paragangliomas (VPGLs) are exceptional, particularly when biochemical activity and metastatic nodal involvement coexist. **Case Presentation:** We report a 33-year-old man with a 2-year history of enlarging bilateral neck masses, positive family history, and markedly elevated noradrenaline (6430 pg/mL; reference range <750 pg/mL). Initial CT angiography suggested bilateral CBTs, and Metaiodobenzylguanidine (MIBG) scintigraphy did not demonstrate abnormal adrenal or distant uptake. After alpha- and beta-blockade, staged surgery was performed. Right CBT excision demonstrated metastatic PGL, with two of four lymph nodes positive. Subsequent MRI and operative reassessment revealed synchronous bilateral CBTs and bilateral VPGLs. Left-sided surgery required partial debulking of a vagal-adherent mass to preserve nerve continuity; pathology confirmed PGL with Ki-67 index approximately 2% and left neck nodes were negative. Postoperatively, profound bradycardia required temporary then permanent pacing, together with bilateral vocal cord paralysis. During follow-up, swallowing and voice improved, the pacemaker was removed, and late imaging showed stable residual cervical disease without visceral metastasis on chest/abdominal CT. **Conclusions:** This case highlights the diagnostic and therapeutic complexity of multicentric, biochemically active HNPGLs and supports individualized multidisciplinary management, genetic counseling, biochemical surveillance, and long-term follow-up.

## 1. Introduction

Paragangliomas (PGLs) are rare, highly vascular neuroendocrine tumors arising from extra-adrenal paraganglia derived from the embryonic neural crest [1]. They belong to the broader pheochromocytoma-paraganglioma (PPGL) spectrum, which includes adrenal pheochromocytomas and sympathetic or parasympathetic extra-adrenal PGLs. Head and neck PGLs (HNPGLs) account for approximately 3% of all PGLs and predominantly originate from the parasympathetic paraganglia, most frequently at the carotid body, followed by the jugular bulb, tympanic plexus, and the ganglion nodosum of the vagus nerve [1,2]. Clinically, carotid body tumors (CBTs) are often indolent and present as slow-growing, painless lateral neck masses; however, proximity to major vessels and lower cranial nerves may create substantial diagnostic and therapeutic complexity even when lesions are histologically low grade [3,4].

Modern PPGL management increasingly emphasizes systematic endocrine and genetic assessment because HNPGLs may be hereditary, multicentric, metachronous, associated with pheochromocytoma or extra-head-and-neck PGLs across the lifespan, and occasionally catecholamine-secreting [5,6,7,8]. Plasma-free or urinary fractionated metanephrines are recommended as initial biochemical tests in suspected PPGL, and functional tumors require alpha-adrenergic blockade before surgery [9]. Although many parasympathetic HNPGLs are clinically nonsecretory, recent data indicate that clinically meaningful catecholamine secretion may be present in approximately 7.7% to 9.2% of HNPGLs, particularly among CBTs, cervical sympathetic PGLs, and SDHB-associated disease [6]. Therefore, characterization of multifocal HNPGL should include anatomical imaging, biochemical assessment, hereditary-risk evaluation, and consideration of whole-body functional imaging when multifocal or metastatic disease is suspected [3,10,11].

Synchronous involvement of CBTs and vagal PGLs (VPGLs) in the same patient remains rare, and reports combining bilateral CBTs with bilateral VPGLs are exceptional [12,13]. The objective of this report was to describe a rare case of synchronous bilateral CBTs and bilateral VPGLs with biochemical activity, histologically confirmed regional nodal metastasis, severe postoperative bradyarrhythmia, and delayed functional recovery, and to contextualize this case against the available literature on multicentric cervical PGLs.

## 2. Case Presentation

### 2.1. Initial Presentation and Diagnosis

A 33-year-old man with no history of chronic medical illness presented with bilateral neck masses that had gradually increased in size over 2 years. There was no history of preceding hypertension, sustained arrhythmia, flushing, carcinoid-like symptoms, collapse/syncope, or hyperadrenergic crises before the initial imaging assessment. A positive family history was recorded, with relatives reported to have had neck masses treated by different methods, although no definite diagnosis had been established from the available family records. On 8 March 2020, initial diagnostic evaluation with computed tomography (CT) angiography, before catecholamine testing was completed, demonstrated bilateral enhancing lesions/tumors arising at the bifurcation of both carotid arteries, encasing the internal carotid arteries and splaying the external carotid arteries bilaterally (Figure 1). No contrast-associated adrenergic crisis occurred during or after CT angiography. The right-sided lesion measured approximately 6.5 × 4 cm and showed some necrotic change, whereas the left-sided lesion measured approximately 6.4 × 3.7 cm. At the time of the initial assessment, these lesions were considered CBTs. Given the potential for a functional PGL, biochemical screening was conducted. Plasma catecholamine levels were markedly elevated, with a noradrenaline level of 6430 pg/mL (reference range <750 pg/mL). Plasma free or urinary fractionated metanephrines, normetanephrines, methoxytyramine, dopamine, serotonin, and 5-hydroxyindoleacetic acid were not measured. A subsequent Metaiodobenzylguanidine (MIBG) scan was performed to screen for adrenal, extra-adrenal, or distant metastatic disease, which showed no evidence of distant metastases at that time.

### 2.2. Pre-Operative Management and First Surgery

The patient was evaluated by the endocrinology team and underwent preoperative medical preparation. Prazosin was administered for alpha-adrenergic blockade before atenolol was added for rate control after alpha blockade had been initiated. This sequencing was used to reduce the risk of intraoperative hypertensive crisis in the setting of biochemical catecholamine excess. After an adequate period of medical preparation, a staged surgical approach was planned, beginning with the slightly larger right-sided tumor. Intraoperatively, the circumscribed tumor was identified at the carotid bifurcation. Multiple enlarged regional lymph nodes were also noted and excised. The procedure was completed without immediate neurovascular complications. Histopathological examination of the resected specimen confirmed PGL, with clear surgical margins and no capsular invasion. Lymph node assessment demonstrated metastatic disease, with two of four excised regional lymph nodes positive for PGL. Nodal stations, Ki-67 index, or the complete immunohistochemical panel were not specified in the first surgical pathology report.

### 2.3. Post-Operative Evaluation and Planning for Second Surgery

Following confirmation of histologically confirmed nodal metastatic disease, a staging CT scan of the neck, chest, and abdomen, including the adrenal regions, was performed and it did not identify adrenal lesions or distant visceral metastases in these regions. Retrospective review of the CT imaging, including coronal reconstructions, clarified the right-sided anatomy as two adjacent right parapharyngeal/cervical masses separated by a visible cleavage plane, rather than a single confluent lesion. In contrast, on the left side, a single mass was identified without a separating cleavage plane. Interval reassessment with magnetic resonance imaging (MRI) of the neck on 7 October 2020, demonstrated no substantial interval change in the cervical disease burden compared with prior imaging. The right-sided lesion measured 3.8 × 3.2 × 3.1 cm, and the left-sided lesion measured approximately 5 × 3.3 × 2.7 cm (Figure 2).

The right-sided tumor indented the right hypopharynx and caused slight narrowing of the lumen at the level of the oropharynx. Multiple signal-void areas were present within the tumor, attributed in the report to prominent vessels. Diffusion imaging demonstrated multiple slightly prominent lymph nodes in the posterior triangle with post-contrast enhancement. The MRI also described a small oval T2-hyperintense focus in the right side of the T2 vertebral body, measuring approximately 4 × 34 mm, which did not suppress on fat-saturated images and enhanced after intravenous contrast (Figure 3). The overall impression was bilateral CBTs and bilateral VPGLs with slightly prominent bilateral cervical lymphadenopathy in the deep posterior triangles and a small focal T2 vertebral body lesion that was radiologically suspicious for possible metastasis but was not histologically confirmed.

Initial cross-sectional imaging was interpreted as demonstrating a single large parapharyngeal mass; however, intraoperative findings and subsequent detailed radiologic review established the presence of two anatomically distinct lesions, consistent with synchronous bilateral CBTs and bilateral VPGLs, biochemical activity, and regional lymph node metastasis. On retrospective review, subtle splaying of the carotid bifurcation and a separate cranial extension along the expected course of the vagus nerve were appreciated, although these findings had not been clearly distinguishable preoperatively because of mass effect. The coexistence of bilateral CBTs and bilateral VPGLs was therefore definitively recognized only after integration of operative findings with detailed radiological re-review and MRI. The case was discussed in a multidisciplinary team meeting involving vascular surgery, head and neck surgery, and endocrinology. A decision was made to proceed with surgery for left-sided neck mass excision with modified neck dissection and possible right-sided completion at a later stage. The patient was again prepared for surgery and was given the same antihypertensive medications.

### 2.4. Second Surgery and Post-Operative Course

On 12 October 2020, the patient underwent left-sided carotid mass excision with neck dissection. Intraoperatively, the tumor was found to be densely adherent to the left vagus nerve. To preserve the continuity and potential function of the nerve, complete resection was not feasible, and a partial debulking of the mass was performed. A full left neck dissection was completed on the left side. Histopathological examination of the specimen confirmed a PGL with a Ki-67 index of approximately 2%. All examined left-sided lymph nodes were negative for tumor involvement (jugular, 0/3; level IIa, 0/1; levels III and VI, 0/12). Postoperatively, the patient was transferred to the intensive care unit (ICU) for monitoring and was initially extubated successfully.

Upon a trial of oral water intake, severe coughing occurred, with no reported aspiration, followed by profound bradycardia and a near-arrest episode that necessitated re-intubation. In the ICU, he experienced multiple episodes of severe bradycardia, with heart rates decreasing to 30 beats per minute. Cardiology consultation was obtained, and a temporary pacemaker was inserted. Bilateral vocal cord paralysis was also documented, likely reflecting vagal-region involvement and surgical manipulation; the left-sided deficit occurred postoperatively, whereas the right-sided deficit was attributed to tumor-related mass effect or prior right-sided surgical manipulation that had not been clinically apparent. His condition gradually improved, and no fever or other apparent signs of infection were documented. Continued cardiology follow-up led to a recommendation for permanent pacemaker insertion, which was subsequently performed. Thereafter, his overall condition improved. He reported some dizziness but was able to walk without severe bradycardia, and his heart rate was documented as 70 beats per minute; he was then transferred to the ward. He was maintained on procyclidine 2.5 mg orally twice daily, later increased to 5 mg orally twice daily. The wound healed without reported complications.

### 2.5. Follow-Up

Follow-up CT of the neck on 19 November 2020 showed that the residual left-sided mass had regressed in size to 3.0 × 1.6 cm after debulking, while the right-sided lesion showed no interval change in size. The study also suggested left vocal cord paralysis, based on medialization of the left vocal cord and an enlarged laryngeal ventricle. Serial postoperative catecholamine or metanephrine testing was not performed after each operation. No postoperative somatostatin receptor imaging with OctreoScan or ^68^Ga-DOTATATE PET/CT was undertaken, and adjuvant octreotide therapy was not initiated. By approximately 1 year postoperatively, the permanent pacemaker had been removed, and the patient reported no further hoarseness or choking episodes. Excision of the right vagal tumor was offered by the head and neck surgeon; however, the patient declined after counseling regarding potential complications. At subsequent follow-up, Magnetic Resonance Angiography (MRA) of the neck on 15 January 2026 reportedly demonstrated no change in size compared with the most recent prior imaging (2023) (Figure 4), and CT of the chest and abdomen showed no evidence of visceral metastasis, as shown in Table 1.

## 3. Discussion

Although many HNPGLs follow an indolent course, their clinical importance lies in their hypervascularity, proximity to major vessels and lower cranial nerves, potential hereditary basis, occasional catecholamine secretion, and the functional consequences of treatment [3,14]. Multicentricity has been reported in 10% to 20% of sporadic cases and may approach 80% in familial PGL syndromes [15,16,17]. Within this spectrum, synchronous CBTs and vagal involvement in the same patient is distinctly uncommon, and the literature remains dominated by isolated case reports rather than robust comparative series (Table 2) [12,13,18]. Therefore, the present case is notable because it combined multicentric bilateral cervical distribution, biochemical activity, histologically confirmed regional nodal metastasis, complex operative anatomy, and severe postoperative cardiolaryngeal morbidity.

Current evidence supports a hereditary framework for a substantial proportion of HNPGLs, and guideline-oriented studies recommend genetic counseling and consideration of germline testing for all patients with PGL, particularly when the disease is bilateral, multifocal, metastatic, or presents at a relatively young age [3,28]. Up to 40% of HNPGLs are associated with genetic mutations, predominantly in the succinate dehydrogenase (SDH) complex genes (SDHA, SDHB, SDHC, SDHD) [3,29,30]. SDHD mutations are classically associated with multifocal parasympathetic HNPGL and demonstrate a parent-of-origin effect, with disease manifesting predominantly after paternal inheritance. In contrast, SDHB mutations are more often associated with extra-adrenal sympathetic PGLs and a higher risk of metastatic disease than SDHD-associated tumors [31,32,33]. In the large Papaspyrou series of 175 patients, multifocality and mutation-positive disease were important recurring themes in clinical evaluation [17]. More recently, Bellamkonda et al. analyzed 49 patients with bilateral HNPGLs comprising 116 tumors, including 90 CBTs and 15 VPGLs, further highlighting that bilateral disease is a distinct management subset [34]. These observations are highly relevant to the present patient because he presented at 33 years of age with bilateral cervical masses and a positive family history of relatives with neck masses. Although formal molecular testing had not been performed, this phenotype strongly supports referral for genetic counseling, germline testing, family evaluation, and long-term surveillance [35].

HNPGLs typically present as slow-growing, painless, pulsatile lateral neck masses. Symptoms often arise from the mass effect on adjacent lower cranial nerves (CN IX-XII), leading to hoarseness, dysphagia, or Horner’s syndrome [2]. The present patient had a 2-year history of progressively enlarging bilateral neck masses, consistent with the slow growth pattern reported for these tumors (mean growth rate of 1–2 mm/year) [36]. Most HNPGLs have historically been considered non-secretory; however, contemporary data indicate that clinically meaningful catecholamine excess is not negligible [6,28,37]. Smith et al. reported that clinically significant catecholamine secretion was present in 9.2% of patients and 7.7% of tumors, with enrichment among CBTs and SDHB-associated disease [6]. This finding is clinically important because biochemical activity influences perioperative preparation, cardiovascular risk, and interpretation of symptoms [38]. In the current patient, the markedly elevated noradrenaline level justified preoperative alpha- then beta-blockade and distinguishes this case from many published HNPGL reports in which biochemical activity was absent or not reported.

The anatomical distribution and size of HNPGLs dictate the surgical approach and associated morbidity. This case featured bilateral lesions initially thought to be isolated CBTs. However, intraoperative findings and retrospective imaging review revealed a complex combination of bilateral CBTs and bilateral VPGLs. CBTs characteristically arise at the carotid bifurcation, splay the internal and external carotid arteries (Lyre sign), and are graded by the Shamblin classification according to carotid vessel encasement [39]. VPGLs typically originate more cephalad from the nodose ganglion and displace the internal carotid artery anteriorly [12]. The synchronous presence of four anatomically distinct parapharyngeal and cervical tumors suggests marked multifocality, a phenotype reported in SDHx-associated disease [4].

Standard anatomical evaluation relies on contrast-enhanced CT and MRI, which define carotid bifurcation splaying, cranial extension, skull-base proximity, and vascular encasement [3,37]. However, in multifocal or closely adjacent lesions, anatomical distinction may be difficult when one tumor distorts the tissue planes of another. In the present case, the diagnosis of synchronous bilateral CBTs and bilateral VPGLs was established only after integration of operative findings, MRI, and detailed radiological re-review. The modern diagnostic pathway has also evolved beyond anatomic imaging alone. Lin et al. recommended ^68^Ga-DOTATATE PET/CT as first-line functional imaging when multifocal or metastatic disease is suspected [3]. Janssen et al. demonstrated that ^68^Ga-DOTATATE PET/CT localized more HNPGL lesions than conventional anatomical imaging and other functional modalities in parasympathetic HNPGLs [10]. Although MIBG scintigraphy did not demonstrate adrenal or distant uptake at initial work-up, current evidence supports ^68^Ga-DOTATATE PET/CT as the preferred functional imaging modality when multifocal or metastatic HNPGL is suspected [11].

Multifocality should not be equated with metastatic behavior. In PPGL, metastatic disease is defined by tumor presence in sites lacking normal paraganglionic tissue rather than by local invasion or histological features alone [3,37]. Clinically evident metastatic behavior occurs in approximately 5% of CBTs, whereas VPGLs appear to carry a higher metastatic propensity, with reviews citing metastatic rates of roughly 10% to 19% [37,40]. In the present case, PGL involvement in two of four regional lymph nodes after the first operation established histologically confirmed regional metastatic disease. By contrast, the T2 vertebral focus was radiologically suspicious but was not biopsy-confirmed; therefore, it should be regarded as a possible, rather than definitive, distant metastasis. This distinction differentiates the current patient from several comparator reports describing combined CBTs/VPGLs without documented nodal metastatic spread [12,13,41]. Long-term survival for metastatic HNPGLs is variable; lymph node metastases may follow an indolent course, whereas distant metastases involving bone or lung may be associated with worse outcomes and may require consideration of systemic or radionuclide-based therapies in selected patients [42].

Management of HNPGLs has shifted from a uniformly aggressive surgical approach toward individualized decision-making that balances tumor control against long-term function [14]. Factors influencing treatment choice include age, comorbidity, tumor size and site, bilaterality, growth, secretory status, baseline cranial nerve function, metastatic behavior, and patient preference [14,28]. CBTs and VPGLs do not carry equivalent operative risk. In the bilateral HNPGL series by Bellamkonda et al., CBTs had the lowest risk of postoperative cranial nerve deficits after surgery compared with vagal and jugular lesions [34]. Del Guercio et al. reported preoperative cranial nerve palsy in 36% of patients, new postoperative nerve dysfunction in 16%, and persistent deficits in 12% in a mixed cervical series managed at an experienced center [43]. For catecholamine-secreting tumors, preoperative alpha-adrenergic blockade before beta-blockade is recommended to reduce perioperative adrenergic risk [9]. For bilateral tumors, concurrent bilateral resection is generally avoided because of the risk of bilateral lower cranial nerve palsy and baroreflex failure [44]. The staged, single-center multidisciplinary strategy used in the present case, with right-sided resection performed first and left-sided surgery undertaken subsequently after recognition of dense vagal adherence, is consistent with a function-conscious approach in bilateral or multicentric HNPGLs [45]. Complete resection was not pursued at the cost of definite nerve sacrifice; instead, partial debulking was undertaken to preserve nerve continuity. This approach aligns with literature supporting selective resection, radiotherapy, or surveillance in anatomically high-risk lesions rather than routine radical excision [28,40]. Functional morbidity remains one of the most important determinants of outcome in HNPGL management, especially when the vagus nerve is involved. Lower cranial nerve dysfunction may manifest as hoarseness, dysphagia, aspiration risk, shoulder dysfunction, or the need for voice and swallowing interventions. Neskey et al. emphasized that postoperative outcomes after HNPGL resection should be judged not only by tumor control, but also by functionally meaningful endpoints such as feeding-tube or tracheostomy dependence, need for vocal fold medialization, and cerebrovascular complications [46]. In the current case, the postoperative bilateral vocal cord paralysis and transient swallowing difficulty reflected the recognized functional burden of surgery for vagal-region disease. The most distinctive complication was the severe postoperative bradyarrhythmia requiring temporary and then permanent pacing, followed by later pacemaker removal after recovery. Major bradycardic events of this severity are uncommon in the HNPGL literature but biologically plausible through carotid sinus hypersensitivity, vagal reflex activation, or manipulation of autonomic structures during surgery [47]. Bauer et al. described carotid sinus hypersensitivity after preoperative embolization of a CBT that required temporary pacemaker placement before definitive resection [48], and Duan et al. reported intraoperative cardiac arrest during CBT resection attributable to carotid sinus hypersensitivity [25]. The present case indicates that profound postoperative bradycardia may occur in parallel with vagal-related laryngeal dysfunction after complex HNPGLs surgery and that both complications may improve over prolonged multidisciplinary follow-up.

### Limitations of the Current Case Report

Several limitations should be acknowledged. First, endocrine characterization was incomplete; biochemical evaluation showed markedly elevated noradrenaline, but plasma or urinary metanephrines, normetanephrine, dopamine, methoxytyramine, serotonin/5-hydroxyindoleacetic acid, and serial postoperative biochemical values were not measured. Second, germline testing of the patient and endocrine/genetic evaluation of relatives were not performed despite young age, family history, bilateral disease, multifocality, and metastatic nodal involvement. Third, radiological and pathological characterization relied on formal reports because source MIBG images, pathological slides, detailed immunohistochemistry, and pathological micrographs were not provided for publication. Additionally, recognized predictors of difficult resections, such as the distance of the tumor to the base of the skull [49], were not assessed. Fourth, the T2 vertebral lesion was radiologically suspicious but not histologically confirmed. These limitations reinforce the need for comprehensive biochemical testing, genetic counseling, functional imaging, family assessment, and structured long-term surveillance in similar cases.

## 4. Conclusions

This real-world case demonstrates a rare presentation of synchronous bilateral CBTs with bilateral VPGLs in a young patient with positive family history, biochemical activity, histologically confirmed regional nodal metastasis, staged surgical management, severe postoperative bradyarrhythmia, bilateral vocal cord dysfunction, and delayed functional recovery. Several practical messages can be drawn. First, bilateral or multifocal HNPGLs should prompt consideration of hereditary disease, genetic counseling, family evaluation, and long-term surveillance. Second, careful re-review of imaging and, where available, contemporary functional imaging may reveal multicentric disease not fully appreciated on initial assessment. Third, treatment should be individualized, particularly when vagal involvement creates a high risk of permanent lower cranial nerve morbidity. Finally, rare cardiovascular complications such as carotid sinus hypersensitivity or vagally mediated bradyarrhythmia should remain within the perioperative differential in complex HNPGL surgery. Despite the severe presentation, the patient’s subsequent functional improvement, pacemaker removal, stable residual cervical disease, and absence of visceral metastasis on late chest/abdominal imaging suggest a favorable clinical course to date, while continuing to justify prolonged multidisciplinary surveillance.

## Figures and Tables

**Figure 1:**
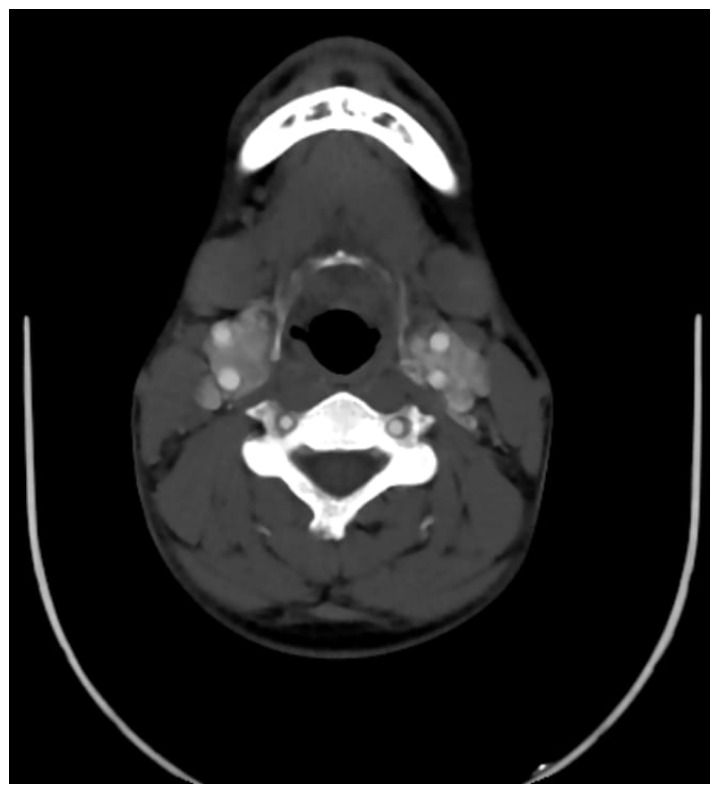
CT angiography of the neck on 8 March 2020 showing bilateral hypervascular carotid bifurcation masses with splaying of the carotid vessels, larger on the right side.

**Figure 2:**
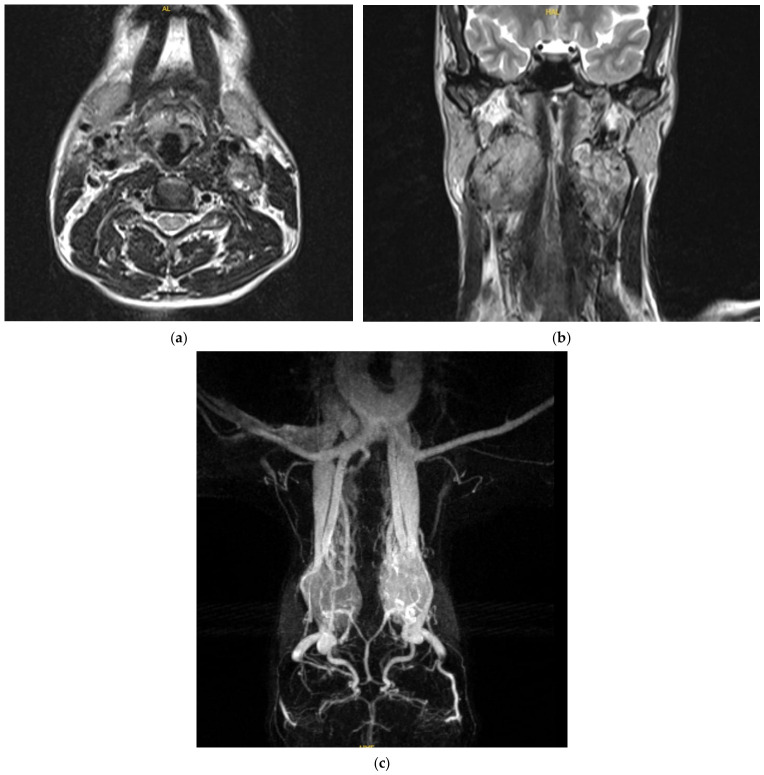
Magnetic resonance imaging (MRI) of the neck demonstrates bilateral paraganglioma-related lesions. (**a**) Axial contrast-enhanced MRI showing bilateral hypervascular masses at the carotid bifurcations with characteristic vascular flow voids. (**b**) Coronal view demonstrating the extent of the lesions and their relationship to adjacent vascular structures, with separation of right-sided lesions suggesting distinct tumors. (**c**) Sagittal view highlighting mass effect on the upper aerodigestive tract, including indentation of the hypopharynx.

**Figure 3:**
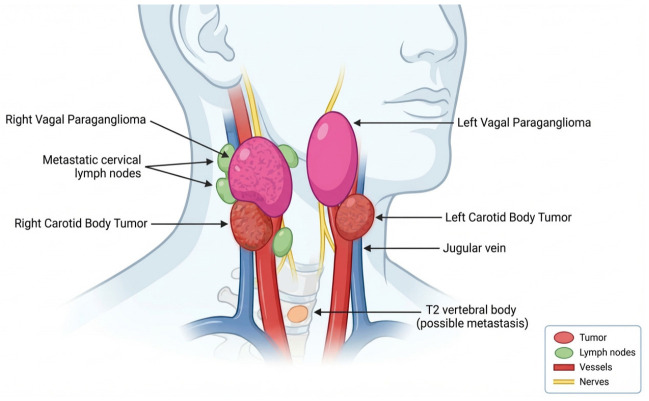
Schematic illustration of the distribution of synchronous bilateral carotid body and bilateral vagal paragangliomas. Enlarged right cervical lymph nodes represent histologically confirmed nodal metastatic involvement, and the T2 vertebral body lesion indicates a radiologically suspected site of possible metastasis. This schematic is not drawn to scale. The schematic was initially hand-drawn by the authors and subsequently enhanced using FigureLabs (https://www.figurelabs.ai/) and BioRender (https://www.biorender.com/). Created in BioRender. Alanezi et.al. (2026) https://BioRender.com/h42n006.

**Figure 4:**
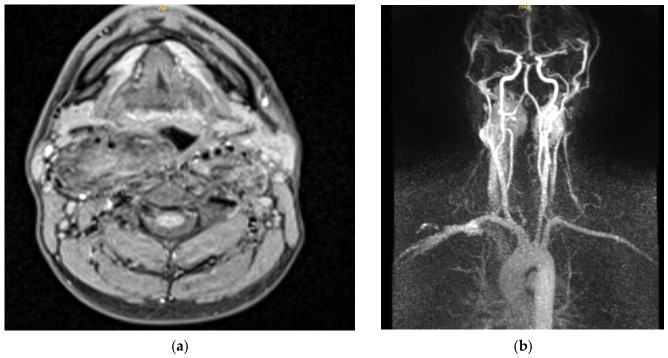
(**a**) Axial Magnetic Resonance Angiography (MRA) of the neck demonstrating postoperative status with no residual tumor at the carotid bifurcation, consistent with complete resection of the carotid body tumor. (**b**) Coronal MRA view showing persistent residual mass along the expected course of the vagus nerve, consistent with residual vagal paraganglioma following prior partial debulking, with no interval progression or new lesions identified.

**Table 1:** Timeline of clinical presentation, diagnostic evaluation, surgical management, pathology, postoperative course, follow-up imaging, and approximate intervals in a patient with bilateral cervical paraganglioma-related masses.

Date/Approximate Interval	Clinical Milestone	Key Documented Details
Before 8 March 2020 (approximately 2-year symptom history before baseline imaging)	Initial presentation	The patient presented with bilateral neck masses that had gradually increased in size over 2 years.
8 March 2020 (baseline imaging)	Initial CT angiography	Bilateral enhancing lesions/tumors were identified at the bifurcation of both carotid arteries, encasing the internal carotid arteries and splaying the external carotid arteries bilaterally.
13 August 2020 (approximately 5 months after baseline CT)	First operation	Excision of a right CBT was performed. Intraoperatively, a large circumscribed CBT with multiple enlarged lymph nodes was identified.
After 13 August 2020 (post-first operation)	First histopathology	Histopathological review, including second-opinion assessment, showed metastatic PGL, with two of four lymph nodes positive for tumor.
7 October 2020 (approximately 8 weeks after first operation)	MRI neck	Bilateral lobulated mass. The right-sided mass indented the right hypopharynx and mildly narrowed the oropharyngeal lumen. Multiple signal-void areas were present within the tumors. Slightly prominent posterior triangle lymph nodes were noted. A small enhancing T2 vertebral body lesion was also reported as concerning for possible metastasis.
After MRI review (October 2020)	Revised anatomical interpretation	Initial cross-sectional imaging had suggested a single large parapharyngeal mass; however, intraoperative findings and subsequent detailed radiologic review established two anatomically distinct lesions, consistent with a CBT and a VPGL.
1 October 2020 (approximately 7 weeks after first operation)	Second admission	The patient was admitted for the second surgery. Hematology, endocrinology, ENT, and cardiology teams were consulted, and he was cleared for surgery.
12 October 2020 (approximately 2 months after first operation)	Second operation	A well-circumscribed tumor at the carotid bifurcation was resected, consistent with a CBT, and a second, anatomically distinct mass was identified that was predominantly adherent to the left vagus nerve. Debulking was performed, and a remnant was left in situ to preserve nerve continuity. Full neck dissection was completed.
After 12 October 2020 (post-second operation)	Second histopathology	Histopathology showed PGL with a Ki-67 index of approximately 2%. All left-sided neck lymph nodes harvested were uninvolved by tumor.
Immediate postoperative period (October 2020)	ICU course	The patient was monitored in the intensive care unit and was initially extubated successfully.
Early postoperative period (October 2020)	Major complication	After drinking water, he developed severe coughing followed by severe bradycardia with near arrest, necessitating reintubation and continued monitoring.
	Cardiac management	Several episodes of bradycardia down to 30 beats/min were documented. Cardiology review was obtained, and a temporary pacemaker was inserted and maintained for several days.
Later postoperative period (late 2020 onward)	Permanent pacing	Cardiology follow-up led to recommendation for permanent pacemaker insertion, which was subsequently performed.
	Recovery status	Clinical condition gradually improved. The patient reported some dizziness but was able to ambulate without severe bradycardia; heart rate was documented as 70 beats/min. The wound was healing well. Procyclidine 2.5 mg orally twice daily was prescribed and later increased to 5 mg orally twice daily.
	Laryngeal complication	Postoperative hoarseness was documented, and bilateral vocal cord paralysis was diagnosed.
19 November 2020 (approximately 5 weeks after second operation)	Follow-up CT neck	There was a reduction in the size of both left- and right-sided lesions compared with the initial imaging, with no residual tumor identified at the carotid bifurcation on either side
2023 (approximately 3 years after surgery)	MRI	The permanent pacemaker was removed, and the patient reported no further hoarseness or choking episodes, with good swallowing.
Jan 2026 (approximately 5 years after surgery)	Late follow-up imaging and staging	MRA neck reportedly showed no change in size compared with the most recent prior imaging (2023), and CT chest/abdomen showed no evidence of metastasis.

CT, computed tomography; ENT, ear, nose, and throat; ICU, intensive care unit; MRI, magnetic resonance imaging; MIBG, metaiodobenzylguanidine; MRA, Magnetic Resonance Angiography; PGL, paraganglioma.

**Table 2:** Published case reports of bilateral or multicentric cervical paragangliomas involving carotid body and/or vagal tumors.

Study; Country	Age/Sex	FHx/Genetics	Functional Status	Presentation	Imaging/Distribution	Metastasis	Management	Complications	Pathology/Outcome
Lemaire et al., 1999; Belgium [19]	24/M	Yes; pedigree suggestive of hereditary PGL with maternal imprinting; pre-SDH linkage only	NR	Initial left cervical swelling	Age 24: left JTP + left CBT; age 26: right CBT + VPGL; age 30: bilateral cervical recurrence with aortopulmonary and mesogastric PGLs	No distant metastasis; multifocal recurrent/systemic disease	Multiple resections	Permanent neurologic sequelae	Histology confirmed PGLs; recurrent metachronous multifocal course
Velegrakis et al., 2001; Germany [20]	75/F	NR	NF biochemically; urinary catecholamines, metanephrines, and VMA normal	Suspected metastatic thyroid carcinoma; rapidly enlarging bilateral lateral neck masses (2 months); no pre-op lower CN deficits	CT: 2 large lateral cervical tumors displacing carotids; right extended to upper mediastinum; left paratracheal thyroid lesion. Intra-op: bilateral vagus nerve encasement	No nodal/distant metastasis from VPGLs	Planned thyroidectomy + bilateral neck dissection; total thyroidectomy; right VPGL en bloc resection; left wedge biopsy only	Persistent hoarseness/right vocal cord paralysis; died POD30 from extensive MI	Right tumor: VPGL with hemorrhage/central necrosis, no atypia or increased mitoses; chromogranin A+. Left biopsy: PGL. Thyroid: papillary microcarcinoma
Pandey et al., 2002; India [21]	23/F	Sporadic; FHx/genetics not detailed	NR	Evening fever, hoarseness, throat foreign-body sensation	Right VPGL + left CBT; CT: bilateral enhancing carotid bifurcation masses; right 4.7 × 2.5 cm with vocal cord involvement and airway narrowing; left smaller	Reactive left level II–III nodes; no distant metastasis	Pre-op lipiodol embolization; staged surgery: right VPGL excision + modified neck dissection, then left CBT excision with partial carotid wall resection/primary repair	After second surgery: PE, ARDS, ventilatory support; died POD4 of multiorgan failure	Biopsy/resection consistent with PGL; NSE positive. Left specimen showed infiltrative nested neoplasm with necrosis; nodes reactive
Karatas et al., 2008; Turkey [13]	49/M	Non-familial	NF	Bilateral neck masses for 3 years with recent tenderness; pulsatile with limited vertical mobility; CN exam initially intact	MRI/Doppler US: vascular masses at both carotid bifurcations (right 3 × 3 × 2 cm; left 4.5 × 3 × 5 cm); incidental left VPGL found intra-op	No nodal/distant metastasis	Pre-op angiography + PVA embolization; staged surgery: left CBT + left VPGL excision first (left vagus sacrificed), then right CBT excision 6 months later	Likely unilateral vocal fold dysfunction; medialization thyroplasty at 4 months	Histology confirmed left CBT + VPGL and right CBT
Kataria et al., 2010; India [12]	20/M	NR	Urinary metanephrine not elevated	Slow-growing bilateral neck masses for 8 years; right neck pain, dysphagia, mouth breathing, hoarseness; left hemitongue atrophy/fasciculations	MRI: right Shamblin III CBT (8 × 6 cm clinically) extending to skull base and compressing oro-/hypopharynx; left VPGL (3 × 2 cm clinically) from carotid bifurcation to skull base with focal dural involvement; bilateral level III/IV nodes; octreotide scan positive	Left VPGL considered malignant with nodal involvement; no distant metastasis	Surgery considered too morbid; IGRT/IMRT 50.4 Gy in 28 fractions to bilateral gross disease/nodes	Grade 2 mucositis during RT	No surgical specimen; 3 months: mild regression/stable disease; 16 months: symptomatic and clinical improvement
Bakshi et al., 2019; India [22]	45/M	FHx not reported; genetics NR	NR	Gradually enlarging left upper-neck mass for 3 years; otherwise asymptomatic	CECT/DSA: bilateral carotid bifurcation lesions with splaying/tumor blush; presumed CBT pre-op; intra-op left carotid + vagal involvement; post-op described as bilateral carotid PGLs with left VPGL	No metastasis	Excision of left carotid and vagal tumors; discharged day 7	Left vocal cord palsy; no aspiration; speech therapy	Otherwise uneventful recovery; no recurrence at 2 years
Yepuri et al., 2021; USA [23]	58/F	Positive FHx (brother with CBT); no germline testing; SDHB IHC loss in thyroid lesion	NR	Presented with thyroid nodule; no palpable cervical masses	Prior right VPGL resection 24 years earlier and right CBT resection 13 years earlier; current US: 24 × 8 × 23 mm vascular isthmus/left-lobe nodule; MRI positive; CT chest/abdomen/pelvis negative	No local recurrence or distant disease	Total thyroidectomy	No surgical complications	Primary thyroid PGL confirmed by pathology/IHC; disease-free at 5 years
Moscona-Nissan et al., 2021; Mexico [24]	61/F	NR	NF	Right pulsatile painless neck mass for 1 year	Angio-CT: right hypervascular carotid-space mass 4.2 × 3.6 × 3.4 cm to C5, encasing proximal ECA; left carotid bifurcation lesion 6.4 × 5.8 mm; right Shamblin II; angiography confirmed tumor blush	No metastasis	Novel pre-op endovascular strategy: covered PTFE stent-graft in right common/proximal ICA + balloon plasty; surgery planned	Arterial spasm/possible distal dissection during procedure; post-procedure MRI/FLAIR negative for ischemia	Histology and long-term surgical outcome not available; short follow-up unremarkable
Duan et al., 2022; China [25]	58/F	FHx negative; genetics NR	NF	Incidentally diagnosed 3 years earlier; later reported only neck-swelling sensation	CTA: bilateral CBTs (right 2.6 × 1.8 × 1.3 cm, Shamblin II; left 1.1 × 0.9 × 0.9 cm, Shamblin I)	No malignancy	Staged surgery planned; right CBT resection first by subadventitial dissection; left observed at reported follow-up; no pre-op embolization or pacemaker	Intra-op asystole/cardiac arrest with hypotension from carotid sinus hypersensitivity; recovered with CPR, atropine, topical lidocaine, and stopping manipulation	Histology/IHC confirmed PGL; no neurologic sequelae at 1 month
Sakaran et al., 2024; Malaysia [26]	NR/M	SDHD mutation positive; FHx NR	NR	Bilateral painless palpable neck masses for 2 years	CECT: bilateral carotid bulb masses with Lyre sign (left 3 × 2.5 cm; right 2 × 1.2 cm); intra-op features consistent with Shamblin II	No metastasis	Staged excision: left CBT then right CBT 3 months later; hypoglossal, ansa cervicalis, and vagus preserved in first surgery	Transient left hypoglossal palsy	NR
Cidral et al., 2025; Brazil [27]	49/F	No FHx; genetics NR	NR	Bilateral neck masses for 1 year with recurrent syncope; no pain, dysphagia, or dysphonia	CTA/angiography: bilateral hypervascular carotid-space masses; left larger; both Shamblin III; cerebral blood-flow study unchanged bilaterally	No nodal/distant metastasis	No pre-op embolization; staged bilateral surgery: right then left 2 months later; subadventitial dissection; blood loss 400 and 600 mL	No postoperative neurologic deficit reported	Histology confirmed bilateral PGLs; article also labeled both grade III, but modern pathology details were not reported

ARDS, acute respiratory distress syndrome; CBT, carotid body tumor; CECT, contrast-enhanced computed tomography; CN, cranial nerve; CTA, computed tomography angiography; DSA, digital subtraction angiography; ECA, external carotid artery; FHx, family history; ICA, internal carotid artery; IHC, immunohistochemistry; IGRT, image-guided radiotherapy; IMRT, intensity-modulated radiotherapy; JTP, jugulotympanic paraganglioma; MI, myocardial infarction; NF, non-functional; NR, not reported; PE, pulmonary embolism; PGL, paraganglioma; POD, postoperative day; PTFE, polytetrafluoroethylene; PVA, polyvinyl alcohol; RT, radiotherapy; SDH, succinate dehydrogenase; VPGL, vagal paraganglioma; VMA, vanillylmandelic acid.

## Data Availability

All data generated or analyzed during this study are included in this published article. Additional details are available from the corresponding author upon reasonable request.

## Abbreviations

The following abbreviations are used in this manuscript:

ARDS	acute respiratory distress syndrome
CBT	Carotid body tumor
CT	Computed tomography
CTA	Computed tomography angiography
MRI	Magnetic resonance imaging
HNPGL	Head and neck paraganglioma
VPGL	Vagal paraganglioma
PGL	Paraganglioma
MIBG	Metaiodobenzylguanidine
VMA	Vanillylmandelic acid
PTFE	Polytetrafluoroethylene
